# Supplementary material for: Polaritonic molecular clock for all-optical ultrafast imaging of wavepacket dynamics without probe pulses
Source: Nat Commun. 2020 Mar 17;11:1423. doi: 10.1038/s41467-020-15196-x (PMC7078293; doi:10.1038/s41467-020-15196-x)
Supplement: Supplementary file 1 — Supplementary Information [file 41467_2020_15196_MOESM1_ESM.pdf]

Supplementary Information: "Polaritonic Molecular Clock for All-Optical Ultrafast Imaging of Wavepacket Dynamics without Probe Pulses"

R. E. F. Silva,[1,*] Javier del Pino,[2] Francisco J. García-Vidal,[1,3] and Johannes Feist[1,†]

[1]*Departamento de Física Teórica de la Materia Condensada and Condensed Matter Physics Center (IFIMAC), Universidad Autónoma de Madrid, E-28049 Madrid, Spain*
[2]*Center for Nanophotonics, AMOLF, Science Park 104, 1098 XG Amsterdam, The Netherlands*
[3]*Donostia International Physics Center (DIPC), E-20018 Donostia/San Sebastián, Spain*

SUPPLEMENTARY NOTE 1 - PHOTODISSOCIATION IN WEAK COUPLING

We here apply the idea of probing the bare-molecule excited-state dynamics in the weak-coupling regime to the case of a molecule undergoing photodissociation. We choose potential energy surfaces similar to those of methyl iodide [1], described by the molecular Hamiltonian

$$H_{\mathrm{m}} = \frac{p^2}{2M} + V_{\mathrm{g}}(q)\sigma^-\sigma^+ + V_{\mathrm{e}}(q)\sigma^+\sigma^-, \qquad (1)$$

$$V_{\mathrm{g}}(q) = D_{\mathrm{e}}\left(1 - e^{-a(q-r_{\mathrm{g}})}\right)^2, \qquad (2)$$

$$V_{\mathrm{e}}(q) = \delta + \frac{\alpha}{q - r_{\mathrm{e}}}, \qquad (3)$$

where $M = 2 \times 10^4$ a.u., $D_{\mathrm{e}} = 2.48$ eV, $a = 0.52$ a.u., $r_{\mathrm{g}} = 7.94$ a.u., $\alpha = 0.11$ a.u., $r_{\mathrm{e}} = 5.48$ a.u., and $\delta = 3.35$ eV. While we worked with mass-weighted coordinates in the main text, q here directly represents the C-I distance. In Supplementary Figure 1(a), we show the potential energy surfaces, which in the weak-coupling regime are not perceptibly modified from the bare-molecule surfaces. However, the strong Purcell effect induces fast radiative decay at the nuclear position where the electronic transition is resonant with the cavity, denoted by $q(\omega_{\mathrm{c}})$. When a laser pulse excites the system on resonance with the molecular Franck-Condon transition, the off-resonant driving leads to transient population of the cavity mode during the pulse, see Supplementary Figure 1(b), and excitation of the molecular excited state. Afterwards, the molecule starts dissociating and, when it reaches $q(\omega_{\mathrm{c}})$, transfers some population to the cavity mode. This in turn emits a short burst of radiation, with the burst duration given by the cavity lifetime. Scanning the cavity frequency then allows to directly reconstruct the dissociation dynamics, as shown by the perfect agreement between the time-dependent radiative emission and the mean nuclear position $\langle q\rangle(t)$ in the excited state (green line) shown in Supplementary Figure 1(b).

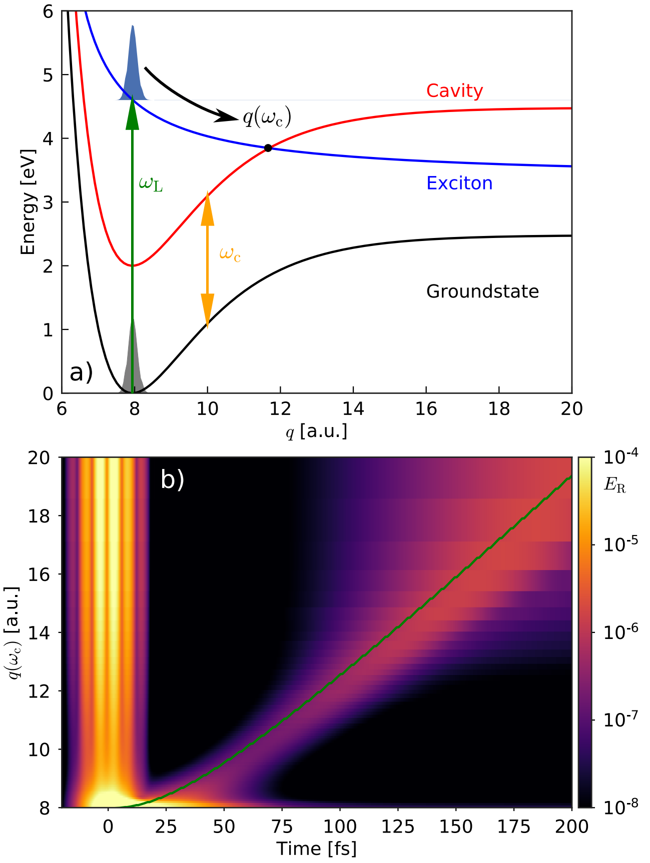

Supplementary Figure 1. **Photodissociation.** (a) PES for a single methyl-iodide-like molecule coupled to a confined light mode, described by its ground (black) and excited (blue) state without photons present, as well as the ground state with a single photon in the cavity (red). The nuclear coordinate where the exciton and cavity PES cross is denoted by $q(\omega_{\mathrm{c}})$. (b) Time-dependent radiative emission E_{R} for $\omega_{\mathrm{L}} = 4.6$ eV, $\Omega_{\mathrm{R}} = 0.05$ eV and different values of the cavity frequency ω_{c}. For all calculations, $E_0 = 2.1 \times 10^{-7}$ a.u. and $\sigma_{\mathrm{L}} = 0.1$ eV. The green line shows $\langle q\rangle(t)$ when no cavity is present.

SUPPLEMENTARY NOTE 2 - TENSOR NETWORK AND THE ROLE OF VIBRATIONAL DEPHASING

In this section, we show that the polaritonic molecular clock approach to probing nuclear dynamics also works when taking into account the many vibrational modes present in a typical organic molecule. The presence of many modes, as well as their coupling to bath phonons in the host matrix, lead to fast vibrational dephasing which is not taken into account in the single-mode approximation employed in the main text. We thus compare these model calculations with calculations performed

using time-dependent variational matrix product states (TDVMPS) approach [2, 3] in which the full phononic spectral density, describing all vibrational modes of the molecule and surroundings, is taken into account. We here provide a short overview of the method, and refer the reader to [2–4] for a more extensive discussion. The full Hamiltonian that we solve is given by

$$H_{\mathrm{TN}}(t) = \omega_{\mathrm{c}} a^\dagger a + \mu_{\mathrm{c}} E(t)(a^\dagger + a) + \sum_{i=1}^{N} \omega_{\mathrm{e}} \sigma_i^+ \sigma_i^- + \frac{\Omega_{\mathrm{R}}}{2\sqrt{N}}(a^\dagger \sigma_i^- + a\sigma_i^+) + \sum_{i=1}^{N} \sum_{k} \left[\omega_{i,k} b_{i,k}^\dagger b_{i,k} + \lambda_{i,k}(b_{i,k}^\dagger + b_{i,k})\right] + \sum_{p} \left[\omega_p f_p^\dagger f_p + \eta_p (f_p^\dagger a + a^\dagger f_p)\right], \quad (4)$$

where N is the number of molecules, $b_{i,k}$ is the bosonic annihilation operator for vibrational mode k on molecule i (with frequency $\omega_{i,k}$), and $\lambda_{i,k}$ is the exciton-phonon coupling strength for each mode. The properties of the vibrational modes are fully encoded by the spectral density $J_{\mathrm{v}}(\omega) = \sum_k \lambda_k^2 \delta(\omega - \omega_k)$. The vibrational parameters were obtained at the TDA-B3LYP level of theory using Gaussian 09 [5]. Since the TDVMPS approach is wavefunction-based, we additionally include a bath of free-space photon modes described by annihilation operators f_p and spectral density $J_r(\omega) = \sum_p \eta_p^2 \delta(\omega - \omega_p)$ for their coupling to the cavity mode. In order to reproduce the exponential decay induced by the Lindblad term representing the cavity losses in the main text (Eq. 6 in the Methods section), we here choose a flat spectral density $J_{\mathrm{r}}(\omega) = \gamma_{\mathrm{c}}/(2\pi)$.

The tensor network approach to quantum dynamics relies on the assumption that the ground-state and low-energy excitations live in a corner of the Hilbert space where entanglement is mainly local (entanglement area law). In this case, a many-component wave function can be efficiently described by a network obtained by contracting many smaller (lower-dimensional) tensors. This fact greatly reduces the needed memory allowing for the computation of a quasi-exact solution of the time-dependent Schrödinger equation for systems where a brute-force approach is impossible [6–9]. In order to obtain a Hamiltonian where this approach is valid, we perform a chain-mapping procedure [10, 11] on the vibrational and free-space photonic modes. After this procedure, each of these baths is described by a single tight-binding chain of harmonic oscillators with nearest-neighbor hopping and only the first chain site (the "reaction coordinate") being coupled to the system. In this form, the Hamiltonian fulfills the requirement of only local interactions in a quasi-1D structure that make treatment in a tensor network form efficient.

In this fully quantum calculation, vibrational dephasing as well as vibration-driven coupling between polariton modes are accurately represented [2]. Interestingly, the chain mapping procedure, which is typically seen as a mostly numerical tool, can also provide direct insight into the dephasing times due to vibrational motion. By comparing the hopping strengths between the reaction coordinate mode and the exciton and the reaction coordinate and the second mode in the chain, one may estimate the importance of additional dephasing due to the presence of more than one vibrational mode. For instance, if the first hopping is much larger than the second one, as it happens in anthracene, a Holstein-type model that takes into account only a single vibrational mode can be expected to capture the relevant physics at short times.

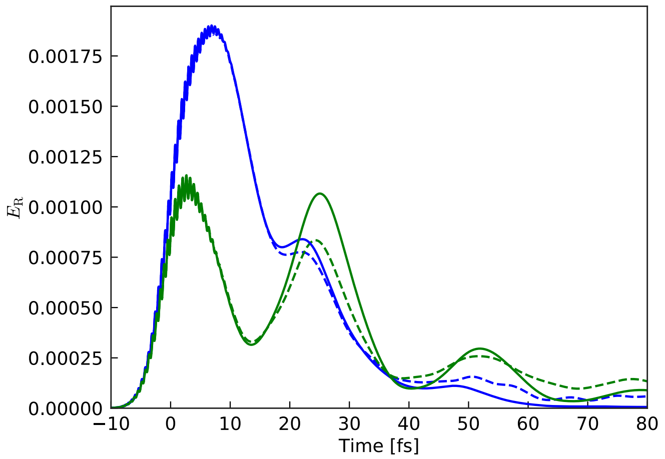

Supplementary Figure 2. **TDVMPS calculations.** Comparison of the radiative emission between the calculations with a single vibrational mode (full lines) and using TDVMPS calculations (dashed lines) in which the full vibrational spectral density of the molecule is taken into account. In green (blue) the frequency of the laser pulse is $\omega_{\mathrm{L}} = 3.3$ (3.7) eV.

This is exactly what we observe in Supplementary Figure 2: the main features of the signal are conserved for short times, but the presence of the additional vibrational modes leads to dephasing of the coherent wavepacket motion and thus suppression of the oscillations on a timescale of tens of femtoseconds. Studying, e.g., the temperature dependence of the dynamics seen here could thus also allow direct insight into vibrational dephasing of molecular excitations and wavepacket motion in a solid-state environment. Moreover, when including several molecules in the model, as shown in Supplementary Figure 3, the signal still presents the signature of the coherent motion of the nuclear wavepackets in all the molecules happening simultaneously. This signature survives even when taking into account the vibrational degrees of freedom of all molecules and the dephasing induced by these. In particular, we find that vibrational dephasing is not significantly more detrimental in the many-molecule case compared to the single-molecule case.

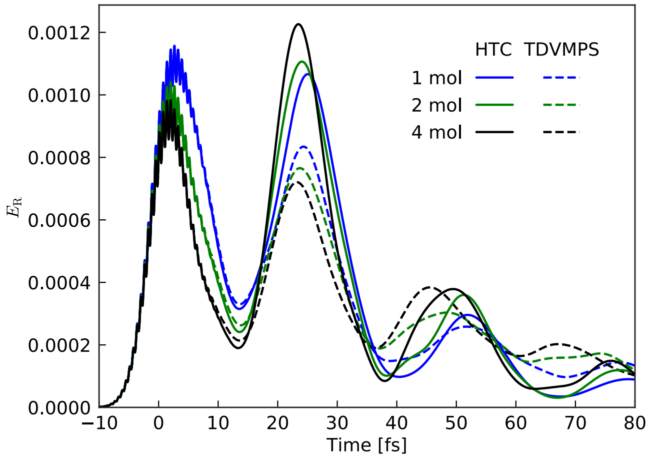

Supplementary Figure 3. **Multiple molecules calculations.** Comparison of the radiative emission between the calculations with a single vibrational mode (full lines) and using TDVMPS calculations (dashed lines) in which the full vibrational spectral density of the molecule is taken into account. In blue/green/black the number of molecules is 1/2/4. The frequency of the laser pulse is $\omega_{\mathrm{L}} = 3.3$ eV.

The height of the first oscillation maximum decreases only slightly when N is increased from 1 to 4. At the same time, this decrease implies that in the macroscopic limit $N \to \infty$, the signal would indeed disappear.

SUPPLEMENTARY REFERENCES

* ruiefdasilva@gmail.com

† johannes.feist@uam.es

[1] Corrales, M. E. *et al.* Control of Ultrafast Molecular Photodissociation by Laser-Field-Induced Potentials. *Nat. Chem.* **6**, 785–790 (2014).

[2] del Pino, J., Schröder, F. A. Y. N., Chin, A. W., Feist, J. & Garcia-Vidal, F. J. Tensor Network Simulation of Non-Markovian Dynamics in Organic Polaritons. *Phys. Rev. Lett.* **121**, 227401 (2018).

[3] Schröder, F. A. Y. N., Turban, D. H. P., Musser, A. J., Hine, N. D. M. & Chin, A. W. Tensor Network Simulation of Multi-Environmental Open Quantum Dynamics via Machine Learning and Entanglement Renormalisation. *Nat. Commun.* **10**, 1062 (2019).

[4] del Pino, J., Schröder, F. A. Y. N., Chin, A. W., Feist, J. & Garcia-Vidal, F. J. Tensor Network Simulation of Polaron-Polaritons in Organic Microcavities. *Phys. Rev. B* **98**, 165416 (2018).

[5] Frisch, M. J. *et al.* Gaussian 09, Revision E.01. Gaussian, Inc (2009).

[6] Prior, J., Chin, A. W., Huelga, S. F. & Plenio, M. B. Efficient Simulation of Strong System-Environment Interactions. *Phys. Rev. Lett.* **105**, 050404 (2010).

[7] Haegeman, J. *et al.* Time-Dependent Variational Principle for Quantum Lattices. *Phys. Rev. Lett.* **107**, 070601 (2011).

[8] Schollwöck, U. The Density-Matrix Renormalization Group in the Age of Matrix Product States. *Ann. Phys.* **326**, 96 (2011).

[9] Chin, A. W. *et al.* The Role of Non-Equilibrium Vibrational Structures in Electronic Coherence and Recoherence in Pigment–Protein Complexes. *Nat. Phys.* **9**, 113–118 (2013).

[10] Chin, A. W., Rivas, Á., Huelga, S. F. & Plenio, M. B. Exact Mapping between System-Reservoir Quantum Models and Semi-Infinite Discrete Chains Using Orthogonal Polynomials. *J. Math. Phys.* **51**, 092109 (2010).

[11] Chin, A. W., Huelga, S. F. & Plenio, M. B. Chain Representations of Open Quantum Systems and Their Numerical Simulation with Time-Adaptive Density Matrix Renormalisation Group Methods. In *Semiconductors and Semimetals*, vol. 85, 115 (Elsevier Inc., 2011).
